# A scoring system categorizing risk factors to evaluate the need for ventriculoperitoneal shunt in pediatric patients after brain tumor resection

**DOI:** 10.3389/fonc.2023.1248553

**Published:** 2023-10-17

**Authors:** Zhong-Yin Guo, Zi-An Zhong, Peng Peng, Yang Liu, Feng Chen

**Affiliations:** ^1^Department of Neurosurgery, Xiangyang Central Hospital, Affiliated Hospital of Hubei University of Arts and Science, Xiangyang, Hubei, China; ^2^Department of Neurosurgery, Tongji Hospital, Tongji Medical College, Huazhong University of Science and Technology, Wuhan, Hubei, China

**Keywords:** pediatric patient, age, blood loss, midline tumor location, preoperative hydrocephalus, tumor resection, risk factor

## Abstract

**Objectives:**

To develop a scoring system based on independent predictors of the need for ventriculoperitoneal (VP) shunt after brain tumor resection in pediatric patients.

**Methods:**

A total of 416 pediatric patients (≤ 14 years old) with brain tumors who underwent surgery were randomly assigned to the training (n = 333) and validation cohorts (n = 83). Based on the implementation of VP shunt, the training cohort was divided into the VP shunt group (n = 35) and the non-VP shunt group (n = 298). Univariate and multivariate logistic analyses were performed. A scoring system was developed based on clinical characteristics and operative data, and scores and corresponding risks were calculated.

**Results:**

Age < 3 (*p* = 0.010, odds ratio [OR] = 3.162), blood loss (BL) (*p* = 0.005, OR = 1.300), midline tumor location (*p* < 0.001, OR = 5.750), preoperative hydrocephalus (*p* = 0.001, OR = 7.044), and total resection (*p* = 0.025, OR = 0.284) were identified as independent predictors. The area under the curve (AUC) of the scoring system was higher than those of age < 3, BL, midline tumor location, preoperative hydrocephalus, and total resection (0.859 vs. 0.598, 0.717, 0.725, 0.705, and 0.555, respectively; *p *< 0.001). Furthermore, the scoring system showed good performance in the validation cohort (AUC = 0.971). The cutoff value for predictive scores was 5.5 points, which categorized patients into low risk (0-5 points) and high risk (6-14 points) groups.

**Conclusions:**

Our scoring system, integrating age < 3, BL, midline tumor location, preoperative hydrocephalus, and total resection, provides a practical evaluation. Scores ranging from 6 to 14 points indicate high risk.

## Introduction

Brain tumors have the highest morbidity and mortality among pediatric patients with solid tumors throughout all stages of childhood (1, 2). The rapid growth and development of the nervous system in childhood make radiotherapy and chemotherapy relatively contraindicated for children with brain tumors (3). As a result, surgery remains the predominant treatment for pediatric brain tumors (1, 3, 4).

Hydrocephalus is a serious postoperative complication in children with brain tumors, characterized by pathological ventricular expansion and increased intracranial pressure. Its pathogenesis may be related to an imbalance between the production and absorption of cerebrospinal fluid (CSF) (5–7). The incidence of preoperative hydrocephalus in children with brain tumors is approximately 50%, while postoperative hydrocephalus can range from 16% to 35% (7) (8, 9). Hydrocephalus can cause many symptoms and sequalae depending on the age of the child such as speech impairment, neuropsychiatric disorders and life-threatening events (7, 10, 11). Prompt ventriculoperitoneal (VP) shunt placement is typically necessary since hydrocephalus tends to be progressive (7). Therefore, it is crucial to identify the risk factors for postoperative hydrocephalus and provide appropriate treatment.

Several studies have emphasized the significant role of age, preoperative hydrocephalus, total resection, and tumor pathologies to predict postoperative hydrocephalus in children with brain tumors, while inconsistent findings prevented from comprehensively evaluating risks (8, 12, 13). Factors such as limited sample sizes, variations in variables, different tumor locations (supratentorial or infratentorial), varying age definitions (ranging from < 16 to < 20 years old), and differences in statistic methods (univariate or multivariate), might contribute to these inconsistencies (9, 14). Moreover, Hu et al. has made a novel discovery regarding the blood loss (BL) as an independent predictor for hydrocephalus in the children with infratentorial tumors (15). In the present work, we comprehensively involved related variables using multivariate analysis and developed a scoring system to assess the occurrence or progression hydrocephalus that needed a VP shunt in children with brain tumors.

## Methods

### Patients and data

**Figure 1:**
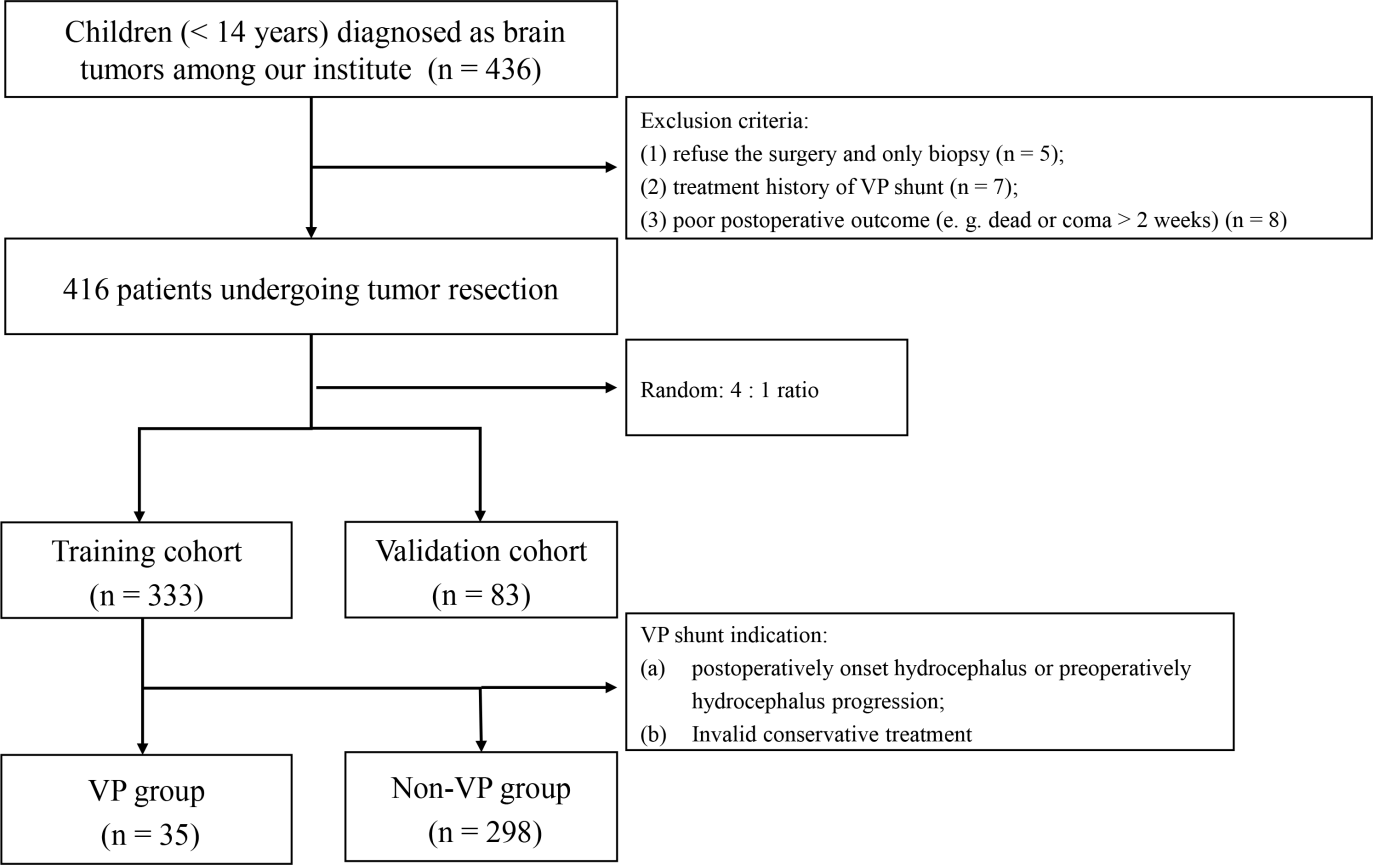
The flow chart of patient selection. VP, ventriculoperitoneal.

**Figure 2:**
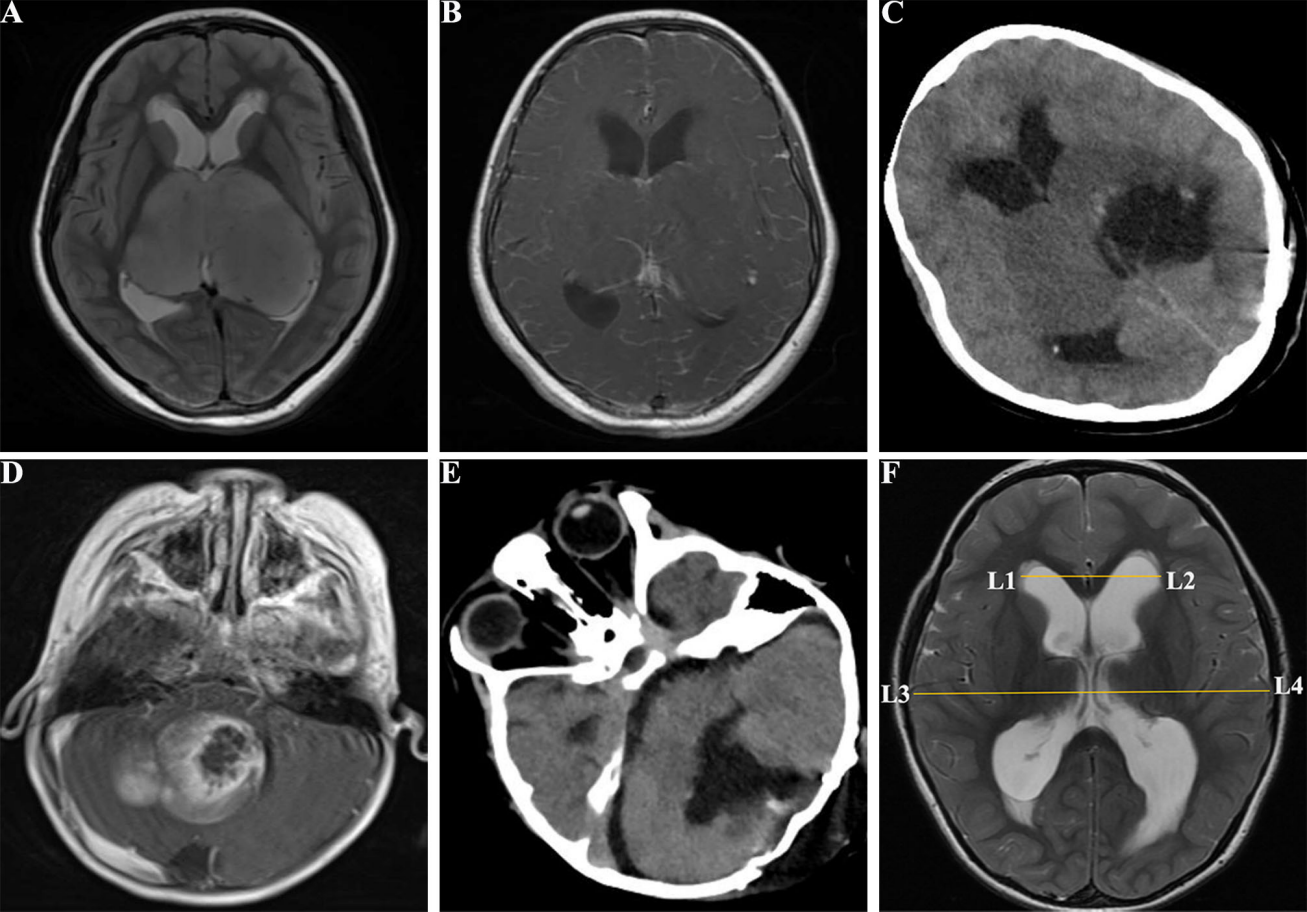
Images of pediatric patients with brain tumors. **(A)**, a 10 years old female with supratentorial tumors in the T2 FSE of MRI before surgery; **(B)**, enhanced MRI T1 FSE+C showed bilateral supratentorial tumors before surgery; **(C)**, CT image of the supratentorial tumor with incomplete total resection; **(D)**, a 3 years old male with infratentorial tumors in the enhanced MRI T1 FSE+C before surgery; **(E)**, CT image of the infratentorial tumor after tumor resection; **(F)**, Postoperative hydrocephalus with Evan’s index > 0.3. FSE, fast spin echo; CT, compute tomography; MRI, magnetic resonance imaging; Evan’s index = L1L2/L3L4.

The flowchart of patient selection is presented in **Figure 1**. The study was approved by Tongji hospital’s institutional ethics committee (TJ_JRB20211271), and data were collected after obtaining consent from the patients’ parents or guardians. From November 2020 to January 2021, a total of 436 patients under 14 years of age were diagnosed with brain tumors and underwent tumor resection at our hospital. Twenty patients were excluded as follows: (a) refusal to undergo surgery or opting for biopsy only (n = 5); (b) previous history of VP shunt treatment (n = 7); (c) poor postoperative outcome, such as death or coma lasting over 2 weeks (n = 8). Next, 416 patients were randomly categorized into the training cohort (n = 333) and validation cohort (n = 83) based on 4:1 ratio. Based on the implementation of VP shunt, the training cohort were divided into a VP group (n = 35) and a non-VP group (n = 298). Hydrocephalus was diagnosed using magnetic resonance imaging, symptoms, and an Evans’ ratio > 0.3 (16) (**Figure 2F**). VP shunts were required for the following indications: (a) postoperative onset of hydrocephalus or progression of preoperative hydrocephalus; (b) failure of conservative treatment.

Clinical characteristics and operative data were collected from medical records in our hospital. The collected variables included operative time, BL (quantified by the intraoperative blood transfusion volume), age (< 3 or ≥ 3 years old), tumor size (≤ 30 mm or > 30 mm), Ki 67 index (≥ 5 or< 5), tumors locations, gender, symptom duration (≤ 1 months or > 1 months), World Health Organization (WHO) grade (I–II [low-grade] and III–IV [high-grade]), presence of preoperative hydrocephalus, extent of tumor resection, American Society of Anesthesiologists (ASA) scale (classified as I–II [low risk] and III–IV [high risk]; patients in V–VI were ineligible to undergo surgery), tumor recurrence, and pathology: medulloblastoma, astrocytoma, ependymoma, and others (primitive neuroectodermal tumor [PNET], oligodendroglioma, choroid plexus papilloma [CPP], choroid plexus carcinoma [CPC], meningioma, neuroblastoma, schwannoma, hemangioblastoma, germ cell tumor [GCT], dysembryoplastic neuroepithelial tumors [DNT], atypical teratoid/rhabdoid tumor [AT/RT], and atypical rhabdomyosarcoma), and infratentorial tumors’ characteristics were outlined in the study conducted by D’Arco F et al. (17). Based on the spatial relationship of tumors to the ventricle and tentorium, tumor locations were classified into two categories: (a) supratentorial (**Figures 2A–C**) or infratentorial tumors (**Figures 2D, E**) as defined by Corti et al. (18); (b) midline tumor location (including tumors located at basal ganglia, diencephalon, third ventricle, lateral ventricles, fourth ventricle, pineal body, cerebellar vermis, and brainstem) or other locations.

### Statistical analysis

Statistical analysis was performed using SPSS 26.0 (IBM Inc, Chicago, IL). Continuous variables were presented as median ± interquartile range, while categorical variables were expressed as frequencies (percentages). The normal distribution of the parameter dataset was assessed using the Kolmogorov-Smirnov test. Univariate logistic analysis was performed to analyze all variables between two groups. Using the stepwise method, significant variables in univariate analysis (operative time, BL, age < 3, Ki-67 index, midline tumor location, infratentorial tumors, WHO grade, preoperative hydrocephalus, total resection, ASA scale, and pathology) were then entered into a multivariate logistic regression (19). A logistic model (Model-Logit) was constructed based on independent risk factors. Risk factor categories were employed to develop a scoring system. Receiver operating characteristic (ROC) curves were generated to calculate significant variables of areas under the curve (AUCs) and cutoffs. The Delong test was performed to compare the AUCs of scoring system in training cohort with in validation cohort. In accordance with the literature, predictive scores and corresponding risk estimate were calculated (20, 21). Differences with *p *< 0.05 were considered statistically significant.

## Results

### Patient demographics

The flow chart of patient selection is shown in **Figure 1**. The training cohort of 333 pediatric patients (< 14 years old) included 196 males and 137 females; 65 patients were under the age of 3, accounting for 20% of the total approximately. Within the training cohort, 173 patients (52%) presented with preoperative hydrocephalus and 299 patients (90%) underwent total resection. Following tumor resection, 35 children underwent VP shunt placement, resulting in an incidence rate of 11%. In the VP group, 5 cases (14%), including 3 children with infratentorial tumors, had obstructive hydrocephalus. The remaining 30 cases (86%) presented with communicating hydrocephalus. The median value of BL was 2.00 U. The pathology of the 155 supratentorial tumors included 84 cases (54%) of low-grade glioma, 15 cases (10%) of ependymoma, 1 case (1%) of medulloblastoma, and 55 cases (35%) categorized as other types including AT/RT, CPP, CPC, cavernous hemangioma, DNT, craniopharyngioma, GCT, meningioma, neuroblastoma, high-grade glioma, PNET, and schwannoma. Among the 178 infratentorial tumors, 73 cases (41%) were diagnosed as medulloblastoma, 59 cases (33%) as astrocytoma, 25 cases (14%) as ependymoma, and 21 patients (12%) presented with other types including PNET, oligodendroglioma, CPP, meningioma, schwannoma, atypical rhabdomyosarcoma, and hemangioblastoma. Besides, 104 cases (31%) were midline tumors. In the VP group, 28 cases (80%), including 2 with postoperative onset hydrocephalus, underwent VP shunt due to hydrocephalus progression within 2 weeks of tumor resection. 3 patients (9%) required the procedure between 2 weeks and 2 months after tumor resection, while two patients (6%) needed it after tumor resection, and another two patients (6%) required it over 2 months later. The demographic differences between the VP and non-VP groups are displayed in **Table 1**.

**Table 1:** Univariate analysis of the predictive factors for VP shunt.

Variables	VP group (n = 35)	Non-VP group (n = 298)	*p* value
Operation time	5.34 ± 2.62	4.55 ± 1.41	**0.001**
Blood loss	3.00 ± 2.00	2.00 ± 1.00	**0.002**
Age			
< 3 y	13 (37%)	52 (17%)	**0.007**
≥ 3 y	22 (63%)	246 (83%)	Reference
Size			
≤30 mm	10 (29%)	109 (37%)	Reference
> 30 mm	25 (71%)	189 (63%)	0.352
Ki 67 index			
≧ 5	23 (66%)	136 (46%)	**0.028**
< 5	12 (34%)	162 (54%)	Reference
Midline tumor location	25 (71%)	79 (27%)	**< 0.001**
Infratentorial tumors	24 (68%)	154 (52%)	0.062
Male	20 (57%)	176 (59%)	0.827
Symptom duration			
≤ 1 months	24 (69%)	207 (69%)	Reference
> 1 months	11 (31%)	91 (31%)	0.914
WHO grade			
I-II	12 (34%)	156 (52%)	Reference
III-IV	23 (66%)	142 (48%)	**0.028**
Preoperative hydrocephalus			
Yes	31 (89%)	142 (48%)	**< 0.001**
No	4 (11%)	156 (52%)	Reference
Total resection			
Yes	28 (80%)	271 (91%)	**0.049**
No	7 (20%)	27 (9%)	Reference
ASA scale			
I-II	24 (69%)	247 (83%)	Reference
III-IV	11 (31%)	51 (17%)	**0.044**
Tumor recrudesce	4 (11%)	35 (12%)	0.956
Pathology			**0.020**
Medulloblastoma	12 (34%)	62 (21%)	**0.004**
Astrocytoma	6 (17%)	137 (46%)	Reference
ependymoma	7 (20%)	33 (11%)	**0.007**
Others	10 (29%)	66 (22%)	**0.021**

Significance level where p < 0.05 were in bold. ASA, American Society of Anesthesiologists; WHO, World Health Organization; VP, ventriculoperitoneal.

### Predictive factors for VP shunt and scoring system

The univariate logistic regression results of the predictive factors for VP shunt are shown in the **Table 1**. To further explore the independent predictors, we used the stepwise forward method to incorporate significant variables in univariate analysis into multivariate analysis, as presented in **Table 2**. The age < 3 (*p* = 0.010, OR = 3.162, CI = 1.314 – 7.608), BL (*p* = 0.005, OR = 1.300, CI = 1.084 – 1.560), midline tumor location (*p* < 0.001, OR = 5.750, CI = 2.406 – 13.742), preoperative hydrocephalus (*p* = 0.001, OR = 7.044, CI = 2.120 – 23.405), and total resection (*p* = 0.025, OR = 0.284, CI = 0.095 – 0.855) were the independent predictors. Based on these findings, we established the Model-Logit and developed a corresponding scoring system, which is presented in **Table 3**. The scoring system provides the corresponding points and risk estimates, as outlined in **Table 4**.

**Table 2:** Results of multivariate logistic regression.

Variables	*β* value	*p-value*	OR value	95% CI
Age < 3	1.151	0.010	3.162	1.314 – 7.608
Blood loss	0.262	0.005	1.300	1.084 – 1.560
Midline tumor location	1.749	< 0.001	5.750	2.406 – 13.742
Preoperative Hydrocephalus	1.952	0.001	7.044	2.120 – 23.405
Total resection	-1.258	0.025	0.284	0.095 – 0.855
Constant	-4.341	< 0.001	0.013	

OR, odds ratio; CI, confidence interval; OR, odds ratio; CI, confidence interval.

**Table 3:** Predictive model using risk factor categories.

Risk factor	Categories	Reference value	W*_ij_*-W*_i_*_REF_	D	Points
Age					
	< 3	1	1	1.151	2
	≥ 3	0 = W_1REF_	0	0	0
Blood loss					
	BL ≤ 2.00	1 = W_2REF_	0	0	0
	2< BL ≤ 4	3	2	0.524	1
	4< BL ≤ 6	5	4	1.048	2
	BL > 6	7	6	1.572	3
Midline tumor location					
	Yes	1	1	1.749	3
	No	0 = W_3REF_	0	0	0
Preoperative Hydrocephalus					
	Yes	1	1	1.952	4
	No	0 = W_4REF_	0	0	0
Total resection					
	Yes	1 = W_5REF_	0	0	0
	No	0	-1	1.258	2

W_ij_, reference value; W_REF_, the basic risk value; D, distance, D = β*(W_ij_ -W_iREF_); Points_i_ = D_i_/B.

**Table 4:** Estimate of risk corresponding to total scores.

Total scores	Estimate of risk	Total scores	Estimate of risk
0	0.48%	8	24.14%
1	0.81%	9	34.96%
2	1.35%	10	47.58%
3	2.26%	11	60.52%
4	3.77%	12	72.13%
5	6.20%	13	81.38%
6	10.04%	14	88.07%
7	15.86%		

### Model-logit and scoring system

The Model-Logit could be established: Logit (P) = -4.341 + 1.151 * age (< 3: yes = 1, no = 0) + 0.262 * BL + 1.749 * midline tumor location (yes = 1; no = 0) + 1.952 * preoperative hydrocephalus (yes = 1, no = 0) – 1.258 * total resection (yes = 1, no = 0). This model was accurate but inconvenient for clinical use. Therefore, we establish a scoring system to assess the need for a VP shunt, whose method is similar to Wilson et al. (20). The scoring system was shown in the **Table 3**. Risk factors were categorized and reference values (W*_ij_*) were set. We set the basic risk value (W*_i_*_REF_) of age < 3, BL, midline tumor location, preoperative hydrocephalus, and total resection as 0, 1U, 0, 0, 1, respectively. When parameters exceeded the W*_i_*_REF_, the greater points represented higher risks. The distance (D) was calculated based on the equation: $D=\beta_{i}*\left(W_{ij}-W_{REF}\right)$ . We set the constant B change of each risk factor for each point in the model. We regarded every increase of 2 U of BL as one point, as follows: B = 2 * β*_BL_*, Points*_j_* = D*_i_*/B. Finally, the risk estimate corresponding to the total score was based on the following equation:

$P=\frac{1}{1+exp\left(-\sum_{i=0}^{p}\beta_{i}\chi_{i}\right)}$ ;

$\begin{array}{c} \sum_{i=0}^{p}\beta_{i}\chi_{i}=\beta_{constant}+\beta_{Age}*W_{1REF}+\beta_{BL}*W_{2REF}+\beta_{TM}*W_{3REF}+\\ \beta_{PH}*W_{4REF}+\beta_{TR}*W_{5REF}+B*Total\;score=0.524*Total\;score-5.337 \end{array}$ . Total cores ranged from 0 to 14 points. The total point and risk estimates are displayed in the **Table 4**.

**Figure 3:**
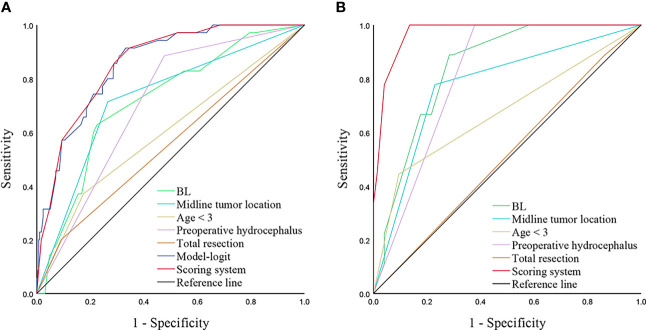
ROC curves analyzing scoring system and independent predictors in training cohort **(A)** and validation cohort **(B)**. A, AUCs of the scoring system, Model-Logit, BL, Midline tumor location, age < 3, preoperative hydrocephalus, and total resection are 0.859, 0.856, 0.717, 0.725, 0.598, 0.705 and 0.555, respectively in the training cohort. B, AUCs of scoring system, BL, Midline tumor location, age < 3, preoperative hydrocephalus, and total resection are 0.971, 0.841, 0.774. 0.675, 0.811, and 0.512, respectively in the validation cohort. ROC, receiver operator characteristic; AUC, area under the curve, BL, blood loss.

To evaluate the performance of our scoring model, we generate ROC curves for independent predictors and models, respectively (**Figure 3A**). Our model demonstrated a significantly higher AUC compared to age < 3, BL, midline tumor location, preoperative hydrocephalus, and total resection (0.859 vs. 0.598, 0.717, 0.725, 0.705, and 0.555, *p* < 0.001, respectively). Besides, AUC of the scoring system was close to Model-Logit (0.859 vs. 0.856, *p* = 0.487). Based on a cutoff value of 5.5 points, the predictive scores classified patients into low-risk (0-5 points) and high-risk (6-14 points) categories. Furthermore, the scoring system demonstrated excellent performance in an independent dataset consisted of 83 pediatric patients with brain tumors (AUC = 0.971) (**Figure 3B**).

## Discussion

Brain tumors are commonly diagnosed in pediatric patients, and surgical resection is the primary treatment (22). However, postoperative hydrocephalus can significantly increase mortality and morbidity, especially in children (15). Previous studies investigating predictors of postoperative hydrocephalus in children with brain tumors have yielded inconsistent findings due to variations in inclusion criteria, statistical methods, limited variables, and sample sizes (1, 9, 12, 23). Most previous studies enrolled patients aged between 16 and 20 years old. However, we believe that including pediatric patients under 14 years old is justified as it allows for a more representative reflection of the patient population, considering tumor spectrum and CSF pathophysiology (15, 24, 25).

In our study, we conducted a comprehensive analysis of correlated parameters in pediatric patients with brain tumors using multivariate analysis. We included patients under 14 years old and analyzed various factors associated with postoperative hydrocephalus. We observed that most cases of postoperative hydrocephalus progression occurred within two weeks. Additionally, we developed a scoring system based on independent risk predictors, including ag e< 3, BL, preoperative hydrocephalus, midline tumor location, and tumor resection. The scoring system exhibited an AUC comparable to that of the Model-Logit (0.859 vs. 0.856, *p* = 0.486) and outperformed any single variable in both the training and validation cohorts.

Consistent with our findings, previous analyses have shown that younger age is associated with a higher risk of postoperative or progressive hydrocephalus requiring a VP shunt (9, 15, 23). The incidence of preoperative and postoperative hydrocephalus is significantly higher in younger children compared to adults (26). Approximately 50% of children are reported to have hydrocephalus at the time of diagnosis, which aligns closely with the rate observed in our training cohort (9). Preoperative hydrocephalus has been found to be significantly associated with the need for VP shunt implementation following tumor resection in children with brain tumors. Surgical trauma, combined with the immature function of CSF circulation, exaggerated intracranial hypertension, and ventricular dilatation, contribute to the increased formation or acute progression of hydrocephalus. Furthermore, the unique anatomical structure of posterior cranial fossa has led to increased interest in exploring the influence of preoperative hydrocephalus on postoperative hydrocephalus in children with infratentorial tumors (9, 23). It is noteworthy that while the incidence of preoperative hydrocephalus is higher in infratentorial tumors compared to supratentorial tumors, the location itself is not significantly associated with postoperative hydrocephalus. It implied another category (midline tumor location and others) may better explain the cause of postoperative hydrocephalus.

Midline tumor location have been identified as an independent predictor of postoperative hydrocephalus formation or progression, which is consistent with previous studies (9, 22, 23). This association may be attributed to the inflammatory reaction of surrounding tissues caused by surgical resection adjacent to the midline. Consequently, adhesion and obstruction of the interventricular foramen, third ventricle, midbrain aqueduct, and fourth ventricle can exacerbate hydrocephalus (27). Additionally, surgical damage to the ventricular zone, blood-brain barrier, and subarachnoid space may contribute to the development or progression of hydrocephalus (27). While the supratentorial or infratentorial categories did not yield significant results, this lack of significance can be partly attributed to the proportion of cerebellar hemisphere tumors. In contrast, the significance of midline tumors underscores its close proximity to the ventricles, subsequently influencing CSF. This observation aligns with existing literature that has emphasized the proximity of infratentorial tumors to the fourth ventricle as a notable risk factor (15). Besides, our data showed that the histology was not an independent predictor of postoperative hydrocephalus. This finding may be explained by the correlation observed between histology and typical midline tumors such as medulloblastoma and ependymoma. Therefore, we recognized the impact of surgical procedures on postoperative hydrocephalus and included the extent of tumor resection in our analyses. Analysis of postoperative images in the VP group revealed that 7 children had not undergone total resection, and among them, 5 cases, including 4 with midline tumors, developed postoperative obstructive hydrocephalus. This underscores the clinical importance of total resection. Furthermore, total resection played an independent protective role, which is consistent with certain studies (14, 22) although some studies have reported conflicting results (9, 15, 23). This discrepancy could potentially be attributed to the limited number of cases involving incomplete total resections.

We introduced a novel predictive variable, BL, which we estimated using intraoperative blood transfusion volume to mitigate the subjective bias of the operator and anesthetist in calculating BL during surgery (15). This approach provides a relatively objective reflection of intraoperative blood volume and maintenance of blood circulation in children. Evaluating intraoperative BL indirectly provides insight into the blood supply, tumor size, and the difficulty of resection, thus offering predictive value for the prognosis of children with brain tumors. Intraoperative hemorrhage can induce an inflammatory reaction and local tissue adhesion in the surgical area. Consequently, this can disrupt the connections between choroid plexus cells and corresponding cells, leading to impaired CSF flow and decreased ventricular volume maintenance function (28). Karimy et al. explored the pathophysiological mechanisms of intraoperative hemorrhage leading to the progression of hydrocephalus and found that hemorrhage can stimulate choroid plexus epithelial cells to produce an inflammatory response through factors like Toll-like receptor 4 and nuclear factor-κB (29). Thus, timely control of bleeding and blood loss management are crucial in children with brain tumors.

## Rationale for scoring system

Although any single markers presented good predictive performance, they were only highlighted by their significance and applied thresholds. Numerous factors contributed to the results. Our scoring system showed the better predictive performance than any single marker both in the training cohort and validation cohort. The risk estimate corresponding to the total point could also be used in future studies. The optimal cutoff value of the scoring system was 5.5 points, which defined patients with low risks (0-5 points) and high risks (6-14 points).

## Limitations of the study

There were several limitations in our study that should be acknowledged. It was retrospective and conducted in a single center, potentially limiting generalizability. Validation using data from other centers would have been preferable. We did not include postoperative CSF tests, and surgical position and imaging characteristics were not accounted for in our analysis. Additionally, BL calculation was challenging due to intraoperative factors, leading us to estimate BL based on transfusion volume. Further research is necessary to validate the findings and address the limitations.

## Conclusions

Most postoperative hydrocephalus progresses within two weeks. The scoring system integrating age < 3, midline tumor location, preoperative hydrocephalus, total resection, and BL could apply practical evaluations. Children with total scores from 6 to 14 points had a high-risk level and need careful attention after surgery.

## Data availability statement

The raw data supporting the conclusions of this article will be made available by the authors, without undue reservation.

## Ethics statement

The studies involving humans were approved by Tongji hospital’s institutional ethics committee. The studies were conducted in accordance with the local legislation and institutional requirements. The human samples used in this study were acquired from primarily isolated as part of your previous study for which ethical approval was obtained. Written informed consent for participation was not required from the participants or the participants’ legal guardians/next of kin in accordance with the national legislation and institutional requirements.

## Author contributions

Z-YG and Z-AZ collected data and wrote the draft. PP: analyzed data. YL and FC: designed, revised, and supervised the study. All authors contributed to the article and approved the submitted version.
